# Ancient interaction between the teneurin C-terminal associated peptides (TCAP) and latrophilin ligand-receptor coupling: a role in behavior

**DOI:** 10.3389/fnins.2015.00146

**Published:** 2015-04-24

**Authors:** Rebecca Woelfle, Andrea L. D'Aquila, Téa Pavlović, Mia Husić, David A. Lovejoy

**Affiliations:** ^1^Department of Cell and Systems Biology, University of TorontoToronto, ON, Canada; ^2^Protagenic Therapeutics Inc.New York, NY, USA

**Keywords:** stress, synapse, molecular evolution, neuroendocrine interactions, adhesion GPCR

## Abstract

Teneurins are multifunctional transmembrane proteins that are found in all multicellular animals and exist as four paralogous forms in vertebrates. They are highly expressed in the central nervous system, where they exert their effects, in part, by high-affinity binding to latrophilin (LPHN), a G-protein coupled receptor (GPCR) related to the adhesion and secretin GPCR families. The teneurin C-terminal associated peptides (TCAPs) are encoded by the terminal exon of all four teneurins, where TCAPs 1 and 3 are independently transcribed as soluble peptides, and TCAPs 2 and 4 remain tethered to their teneurin proprotein. Synthetic TCAP-1 interacts with LPHN, with an association with β-dystroglycan, to induce a tissue-dependent signal cascade to modulate cytoskeletal dynamics. TCAP-1 reduces stress-induced behaviors associated with anxiety, addiction and depression in a variety of models, in part, by regulating synaptic plasticity. Therefore, the TCAP-1-teneurin-LPHN interaction represents a novel receptor-ligand model and may represent a key mechanism underlying the association of behavior and neurological conditions.

## Introduction

The molecular association of adhesion proteins and G protein-coupled receptors (GPCRs) became established in the evolution of eukaryote and later multicellular organisms, and their relationship with energy metabolism would be essential to the survival of these systems. Ultimately, such fundamental molecular transducing systems would have been evolutionarily retained and incorporated into newly evolving signaling and biochemical pathways. The teneurin-latrophilin (LPHN) intermolecular trans-synaptic pairing formed at the earliest stages of metazoan evolution, and is currently the only known example of a trans-synaptic molecular adhesion coupling that is common between invertebrates and vertebrates. However, although the teneurins are well established to play a role in adhesion, these multifunctional transmembrane proteins are also associated, in part with paracrine signaling via a peptide termed “teneurin C-terminal-associated peptide (TCAP),” found in the terminal exon of each of the four teneurin genes. Its primary structure is highly conserved throughout animal evolution and is associated with modulating numerous stress-associated behaviors. In this review, we posit that the teneurin/TCAP-LPHN unit represents one of the most fundamental signaling systems to regulate neuronal metabolism and associated behaviors.

## Discovery and structure of teneurins, TCAP, and latrophilins

The teneurin and LPHN ligand receptor system represents an early evolving system that has become associated with the coordination of cellular development, sensory regulation and control of energy systems to promote the survival of cells in multicellular organisms. On the distal extracellular tip of each teneurin is a short amino acid sequence termed “TCAP” that has actions independent of the full-length teneurin protein. Also, TCAP possesses a compelling structural homology with a number of bioactive peptide ligands associated with the secretin family of GPCRs. The teneurin TCAP protein binds and activates the LPHNs, a family of adhesion- associated GPCRs, to regulate numerous neurological and physiological activities.

### Teneurins

**Figure 1:**
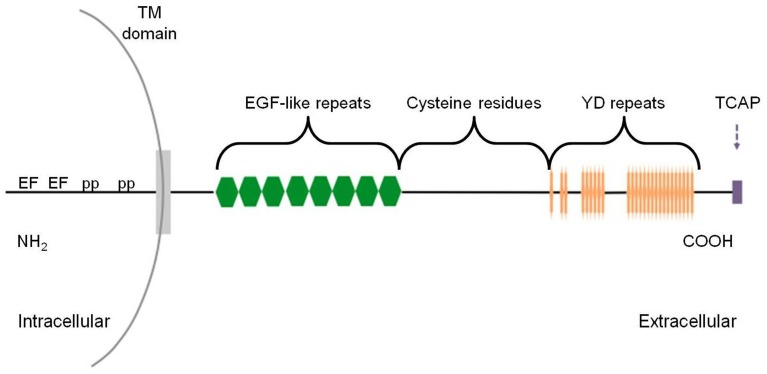
**Schematic of teneurin-TCAP protein structure**. The intracellular amino terminus contains polyproline (pp) sites and EF-hand-like Ca^2+^ binding motifs (EF). The extracellular domain is composed of eight epidermal growth factor (EGF)-like repeats, a cysteine-rich region, followed by 26 tyrosine-aspartic acid (YD) repeats. Finally, the carboxy terminus contains the TCAP structure with 40–41 residues. Drawing is not to scale.

The teneurins (Figure 1) comprise a family of glycosylated type II transmembrane proteins that were originally discovered in *Drosophila* as tenascin-like molecule accessory (ten-a) (Baumgartner and Chiquet-Ehrismann, 1993), tenascin-like molecule major (ten-m) (Baumgartner et al., 1994) and odd oz (odz) (Levine et al., 1994), by two independent groups in a search intended to identify orthologs of the vertebrate tenascins (Baumgartner et al., 1994) and tyrosine phosphorylated proteins (Levine et al., 1994). However, they were eventually established to be structurally and functionally distinct from the tenascins, despite the high degree of conservation of their epidermal growth factor (EGF)-like repeats (Tucker et al., 2006). The name “teneurins” reflects the protein's high level of expression in the developing and adult nervous system, as well as its association with ten-m (Oohashi et al., 1999; Lovejoy et al., 2006). Teneurin genes encode large proteins that are composed of approximately 2800 amino acids and contain an N-terminal intracellular domain, a single span transmembrane domain and a large highly conserved C-terminal extracellular domain (Rubin et al., 1999; Tucker and Chiquet-Ehrismann, 2006), consistent with the architecture of prokaryote polymorphic proteinaceous toxins (see below). The intracellular region contains two EF-hand-like domains, typical of calcium-binding proteins, as well as two polyproline regions which serve as c-Cbl-associated protein/ponsin binding sites, facilitating interaction between teneurin-1 and the cytoskeleton (Nunes et al., 2005). On the highly conserved extracellular side, there are eight tenascin-type EGF-like repeats, a region of conserved cysteine residues, and a unique stretch of 26 tyrosine-aspartate (YD)-repeats (Minet and Chiquet-Ehrismann, 2000; Young and Leamey, 2009). Among eukaryotic proteins, the 26 YD repeats occur only in teneurins.

Subsequently, the vertebrate orthologs of ten-a and ten-m were discovered. Mouse *Doc4*, the first vertebrate member of the teneurin family, was identified in a screen for novel genes that were expressed in response to perturbation of protein folding in the endoplasmic reticulum (Wang et al., 1998) and since then, several laboratories independently described the *ten-a* and *ten-m/odz* homologs in zebrafish (Mieda et al., 1999), chicken (Mieda et al., 1999; Rubin et al., 1999), mouse (Oohashi et al., 1999), rat (Otaki and Firestein, 1999), human (Minet et al., 1999; Minet and Chiquet-Ehrismann, 2000), and *Caenorhabditis elegans* (Drabikowski et al., 2005). In most invertebrates, only one teneurin copy has been identified, with the exception of insects, where two paralogs have been discovered (Tucker et al., 2012). However, unlike invertebrates, four teneurin paralogs have been reported in most vertebrates.

In metazoans, it has been postulated that the teneurins arose from a single ancestral gene. Comparison of the gene organization among human *Ten-1*, *Drosophila ten-a* and *ten-m* and the *C. elegans ten-1* reveals the presence of both conserved intron locations and exon sequences (Minet and Chiquet-Ehrismann, 2000). Sequence comparisons of teneurins show that it is not possible to classify any of the vertebrate teneurins specifically with either *Drosophila ten-a or ten-m*. However, phylogenetic analysis suggests that insects and vertebrates separated before the teneurin gene started to duplicate in each indicating that the two insect *ten-m* (teneurin) homolog genes are the result of a lineage-specific duplication. Therefore, the insect teneurin ancestor gene duplicated once to allow for two teneurin paralogs, *ten-a and ten-m*, whereas the duplication of the teneurins in the vertebrates occurred before the vertebrates radiated into their respective classes.

Recent evidence indicates that the original transfer of teneurin-like homologs originated from a prokaryotic transmembrane polymorphic proteinaceous toxin gene into the genome of a choanoflagellate. Subsequently, it became associated with another gene possessing a set of EGF- like domain repeats, which are a hallmark of metazoan genomes (Tucker et al., 2012; Tucker, 2013). Before the advent of single-celled eukaryotes (protists), prokaryotes were the initial form of life on the planet. Prokaryotes were likely the major food source of the first eukaryotes. As the first heterotrophic (non-phototrophic) multicellular organisms evolved, prokaryotes and protists would have been the fundamental nutrient sources. Choanoflagellates are thought to be the most closely related protist lineage to the Metazoa (Ni et al., 2012; Ramulu et al., 2012; Tucker et al., 2012; Tucker, 2013).

Teneurins and their invertebrate homolog, ten-m, have been found in most metazoans studied to date. In vertebrates, teneurins have been functionally implicated in neurite outgrowth (Rubin et al., 1999), cell adhesion (Rubin et al., 2002), and neuronal pathfinding. In neuroblastoma cells, overexpression of teneurin-2 led to enhanced neurite elongation, enlarged growth cones, increased filopodia formation and co-localization with actin-containing filopodia (Rubin et al., 1999, 2002). Similar effects were also observed in chicken dorsal root ganglia explants plated on recombinant teneurin-1 YD-repeats (Mieda et al., 1999). In mice, a missense mutation at the C-terminus of teneurin-4 results in delayed gastrulation and neural tube defects prior to embryonic lethality (Lossie et al., 2005). Knockout mice lacking teneurin-3 show abnormalities in mapping of ipsilateral projections, indicating that teneurin-3 has a role in axonal guidance within the visual cortex. These mice also exhibit deficits when performing visually mediated behavioral tasks, however the deficits were described as mild, suggesting functional redundancy with other teneurins. Similar actions are observed within the invertebrate teneurins. The *Drosophila* ten-a and ten-m are required for correct matching of olfactory projection neurons and receptor neurons, leading to proper olfactory mapping (Hong et al., 2012). Thus, the conservation of the teneurin gene, its function, and duplication in multicellular organisms argues for its early essential role in species survival and evolutionary success.

### Teneurin C-terminal associated peptide (TCAP)

The discovery of TCAP in rainbow trout occurred after the reports of teneurin and *ten-m* by an independent study, searching for homologs of corticotropin-releasing factor (CRF) (Qian et al., 2004). Alignment with other genomic sequences uncovered a 40-residue carboxy-terminal sequence located in the final 3′ exon of teneurin, now known as TCAP (Qian et al., 2004). Flanked by a cleavage motif on the amino terminus and an amidation motif on the carboxy terminus, TCAP contains features characteristic of an endogenous bioactive peptide (Qian et al., 2004). In comparing teneurin-3 orthologs, a high degree of conservation was observed across the zebrafish, mouse and human, and the TCAP portion embedded within the teneurin carboxy terminal was found to be the most highly conserved sequence of the final exon; this resistance to change is highly suggestive of functional importance. The TCAP sequence alone bore the closest resemblance to the CRF peptide family, in terms of amino acid sequence, than any other known peptide sequences. Since then, it has been shown to possess sequence similarity to all of the secretin GPCR family peptide ligands (Lovejoy et al., 2006; Lovejoy and de Lannoy, 2013). It has since been hypothesized that the original TCAP-like peptide introduced into the Metazoa by lateral gene transfer spawned the evolution of the peptide ligands of the secretin family of GPCRs. This hypothesis is further supported by the close relationship among the cognate receptors (see further discussion below).

Recent studies on the evolution of prokaryote polymorphic proteinaceous toxins indicate that the C-terminal region of the teneurins is derived from the toxin “payload” resulting from the original gene transfer in choanoflagellates (Zhang et al., 2012). This confirmed previous studies on the biological efficacy of the TCAPs. The TCAP family of peptides was first discovered in a search for novel CRF homologs (Qian et al., 2004). Since then, four TCAPs have been identified in vertebrates, annotated as TCAP-1,-2, -3, and -4, based on their location in the four teneurin genes. All TCAPs are the same size as CRF, but possess less than 20% sequence similarity to the CRF family of peptides (Lovejoy et al., 2006) and differ markedly in structure (Tan et al., 2012). However, the TCAP family is highly conserved across the metazoans.

In the genome, TCAPs are annotated as being part of the extracellular domain of teneurin but recent studies indicate that some members of the TCAP family may possess functions that are independent of the larger teneurins. Rodent brain teneurin-1 (Zhou et al., 2003) and TCAP-1 (Wang et al., 2005) mRNA expression is distinct in some regions such as in the limbic areas, but overlap in others areas such as the olfactory bulb and cerebellum suggesting that the teneurin gene could be differentially regulated. Northern blot studies in the adult brain and embryonic hypothalamic cell culture demonstrated that only TCAP-1 and -3 can be independently synthesized from the larger teneurins, (Chand et al., 2012a).

### Latrophilins

A receptor for the teneurins and TCAP has recently been characterized through an independent identification of the black widow spider toxin component, α-latrotoxin. This led to the discovery of the LPHNs, a family of GPCRs (Lelianova et al., 1997). Like the teneurins, recent studies have indicated that the LPHNs, evolved, in part, by lateral gene transmission from prokaryotes to an early metazoan ancestor (Zhang et al., 2012). Based on sequence comparisons of other GPCRs known at the time, LPHN was a novel member of the secretin family of GPCRs. Since then, recent analyses have placed the receptors within the adhesion family of GPCRs (Silva et al., 2011). There are three types of LPHNs (-1, -2, and -3), and both LPHN-1 and -3 are expressed in the brain, whereas LPHN-2 shows expression in non-neural tissues (Silva and Ushkaryov, 2010). The authors suggest that LPHN may play a role in the regulation of the presynaptic release of neurotransmitters in a system that regulates the role of calcium.

LPHNs possess a ligand-binding domain that is characterized as a “hormone binding-domain” as identified by Perrin et al. (1998) based on the characterization of the ligand binding domain of the CRF receptors. This region is found in a number of the adhesion GPCRs (Matsushita et al., 1999; Vakonakis et al., 2008). The toxin, α-latrotoxin, acts a potent agent via its interaction with the hormone-binding domain of LPHN-1 to regulate presynaptic neurotransmitter function by influencing, in part, the voltage-gated calcium channels (Davydov et al., 2009; O'Sullivan et al., 2014). Specifically, α-latrotoxin has been shown to release both γ-aminobutyric acid (Storchak et al., 2002) and acetylcholine (Lelyanova et al., 2009). However, because it produces neurotransmitter release in both calcium containing and calcium free media, it was thought to act on different receptor sites (Storchak et al., 2002). The stimulation of neurons by α-latrotoxin leads to a fusion of synaptic vesicles even in the absence of action potentials, calcium ionophores or hypertonic sucrose media (Boudier et al., 1999; Storchak et al., 2002).

At the time of the LPHN protein discovery, the endogenous ligands were not known, but were later shown to interact with synaptic scaffolding proteins and were generally implicated in presynaptic function leading to neurotransmitter release (Lelianova et al., 1997; Vakonakis et al., 2008; O'Sullivan et al., 2014). The LPHNs, which possess three paralogous genes and proteins and a number of splice variants, were eventually established to be part of the adhesion family of G-protein coupled receptors and were subsequently termed Lec 1, 2, and 3 by virtue of their lectin-like-binding regions (Lelianova et al., 1997; Matsushita et al., 1999). The LPHNs consist of multidomain regions possessing a rhamnose-binding lectin-like domain, an olfactomedin-like domain, and a hormone-binding domain similar to that previously established for the CRF family of receptors (Perrin et al., 1998; Vakonakis et al., 2008). As mentioned previously, it was thought that LPHNs were associated with the Secretin family (Family B) group of GPCRs, as both LPHN and the secretin family both contain a unique hormone-binding domain (Fredricksson et al., 2003; Nordstrom et al., 2009; Schiöth et al., 2010). However, more recently, LPHNs have been found to belong to the Adhesion family of GPCRs (Silva et al., 2011).

Several studies support a significant functional and physical interaction between teneurins and LPHNs, which is integral for the maintenance of the stability of the synaptic cleft. Silva et al. (2011) show that teneurin-2 binds and activates LPHN-1 with a Kd of less than 2 nM. Moreover, a C-terminal fragment of teneurin-2, comprising the region that encompasses TCAP-2 likewise binds LPHN-2 inducing release of intracellular calcium stores. The authors suggest, in the case of teneurin-2, the TCAP-2 region likely remains tethered to the teneurin-2 protein to produce its physiological and behavioral effects. In this study, there was little evidence to suggest that teneurins-1, -3 or -4 bound to the LPHN-1 receptor, and suggest that teneurin-2 was one of the cognate ligands for LPHN-1. However, a later study, confirmed that teneurin-2 did, indeed, bind to LPHN-1 with similar affinity, but teneurin-4 also bound the receptor with a physiologically similar, albeit somewhat lower affinity than teneurin-2 (Boucard et al., 2014). In this study, the lectin domain region of LPHN-1 was essential for full affinity of the teneurin ligand, which is consistent with the lower affinity and activity established for the C-terminal TCAP-2 containing fragment previously reported (Silva et al., 2011). As previously mentioned, the lectin domain is the most highly conserved region of LPHNs, which further supports the ancient ligand receptor interaction and coevolution of teneurin and LPHN.

### Dystroglycans and additional proteins in the teneurin-LPHN complex

**Figure 2:**
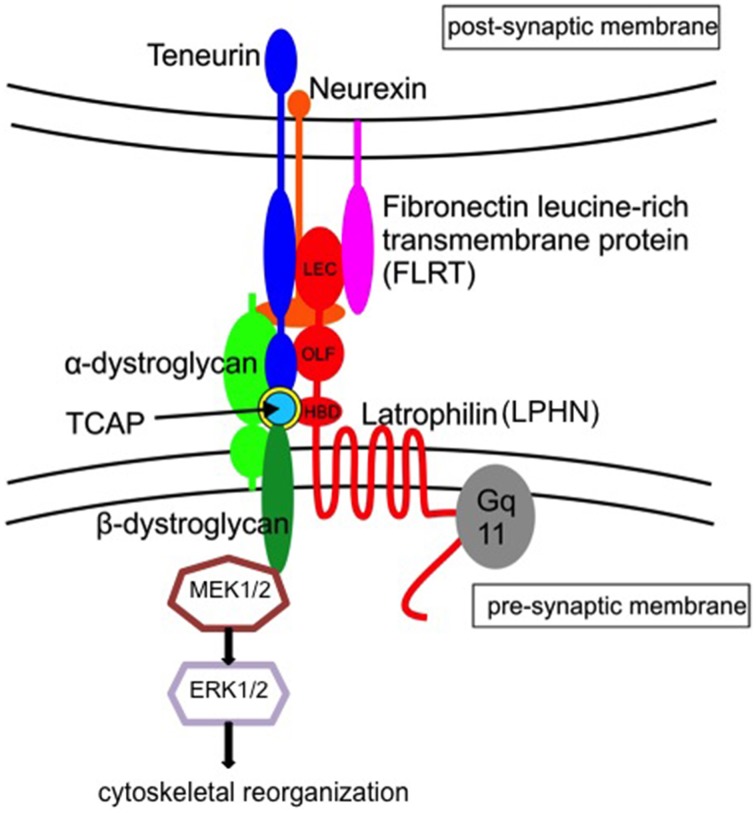
**Schematic interpretation of the teneurin/TCAP-latrophilin complex in the trans-synaptic region**. Current evidence suggests that teneurins interact with both the lectin-like (LEC) and olfactomedin-like (OLF) binding domains. We further postulate that TCAP interacts with the hormone-binding domain (HBD) of latrophilin, and associates with β-dystroglycan to activate the MEK-ERK1/2 pathway leading to cytoskeletal reorganization and synaptic plasticity (Adapted from Chand et al., 2012b). Teneurin/TCAP and latrophilins, together with α–dystroglycan, neurexin and fibronectin leucine-rich transmembrane protein (FLRT) play a role with the stabilization of the trans-synaptic region.

Given the complexity of the teneurin and LPHN proteins, it is unsurprising that the association of these two protein systems results from complex stoichiometry (Figure 2). Additional proteins including dystroglycans, fibronectin leucine-rich transmembrane (FLRT) proteins and neurexins have also been associated with the organization and signaling of this system (Sugita et al., 2001; O'Sullivan et al., 2014). Research shows that the FLRT proteins bind to the lectin domain of LPHNs and are involved in excitatory synapse development, axon guidance, and cell migration in the hippocampus and cortex (O'Sullivan et al., 2012). Moreover, a number of studies indicate a functional and physical association of the teneurin-TCAP protein and dystroglycans. Dystroglycans were initially established as intercellular adhesion proteins in a number of tissues. They arise as single precursor proteins that are then cleaved into α and β forms where they remain associated in the membrane. The α subunit is extracellularly exposed and binds a number of extracellular matrix proteins in non-neuronal cells, including laminin, perlecan, and agrin. Neurexins have been implicated as extracellular binding sites for neuronal α-dystroglycan. Similar to laminin, perlecan, and agrin, the extracellular sequences of neurexins contain a laminin-neurexin-sex hormone-binding/laminin G domain (Sugita et al., 2001). The β-dystroglycan form, on the other hand, consists of a smaller transmembrane region and a cytoplasmic tail. There is little exposure of this subunit to the extracellular space. The intracellular region of the β subunit binds to dystrophin and utrophin, both of which interact with the actin cytoskeleton (Hemler, 1999; Henry and Campbell, 1999). Thus, the dystroglycan complex serves, in part, to couple extracellular matrix proteins with the intracellular matrix. The significance of this arrangement is underscored by deletion studies indicating that loss of the dystroglycan gene results in embryonic lethality (Henry and Campbell, 1998).

Dystroglycan is a main component of the transmembrane protein complex, dystrophin-glycoprotein complex, originally discovered in skeletal muscle (Masaki and Matsumura, 2010). In muscle, dystroglycan acts as a scaffold providing a crucial link between extracellular matrix and the cytoskeleton (Henry and Campbell, 1996; Masaki and Matsumura, 2010), and is also present at the neuromuscular junction where it links rapsyn and acetylcholine receptor, thereby aiding in acetylcholine receptor clustering (Banks et al., 2003). Mice that are homozygous null mutants for dystroglycan result in embryonic lethality, which indicates it is critical for development (Williamson et al., 1997). However, dystroglycan is expressed in non-muscular tissue as well and has functional properties that are not muscle-specific (Henry and Campbell, 1996). Dystroglycan plays an important role in kidney epithelial morphogenesis by its interaction with laminin (Henry and Campbell, 1996), as well; neuronal dystroglycan is responsible for the maintenance of Schwann cell myelination and axonal guidance (Masaki and Matsumura, 2010).

In Trzebiatowska et al. (2008) initially reported an association of the teneurins with dystroglycans. It was concluded that the teneurins have partly related and redundant functions in *C. elegans* development, particularly with respect to the organization and maintenance of the basement membrane in tissues associated with the gonad, pharynx and hypodermis. Both teneurins and dystroglycans bind laminins (Rubin et al., 1999; Henry et al., 2001), a key component of the basement membrane and extracellular matrix in non-neural tissues. Although these studies pointed to a functional relationship between teneurins and dystroglycans, a physical association was not established. In an independent study, Chand et al. (2012b) identified α-dystroglycan as a candidate target for further investigation, when it was found to have the highest level of mRNA up-regulation as ascertained by gene array studies following the treatment of synthetic mouse TCAP-1 on cultures of immortalized mouse hypothalamic cells. It was subsequently determined that fluoresceinisothiocyanate (FITC)-labeled mouse TCAP-1 with lysine substitutions for arginine residues 8 or 37 (FITC-K8/37-mTCAP-1) showed significant co-localization with immunoreactive β-dystroglycan although an association with α-dystroglycan was equivocal. However, although FITC-K37-mTCAP-1 was internalized into the cell via clathrin-independent but caveolin-associated vesicle transport, no co-labeling of this TCAP-1 analog with either of the dystroglycan subunits could be discerned (Chand et al., 2013). These findings were partially resolved when TCAP-1 and teneurin-1 was investigated in the mouse testes. Here, the studies indicated a clear relationship of teneurin-1 with α-dystroglycan, and TCAP-1 with β-dystroglycan (Chand et al., 2014). However, in these studies, teneurin immunoreactivity was found at the cell membrane, whereas TCAP-1 immunoreactivity was typically found within the cytosol. Thus, it was not clear if the intact teneurin-TCAP protein was binding to both dystroglycan subunits. Although these studies show a different interaction profile of the teneurins and TCAPs with respect to dystroglycans, it is not clear whether a similar arrangement occurs in the brain. Thus, based on these studies, the authors suggested that while the dystroglycans were associated with the teneurins and TCAPs, they were not the cognate receptors.

In the brain, the arrangement of the dystroglycan complex with components of the extracellular matrix differs as laminin, perlecan, and agrin are not typically found (Sugita et al., 2001). Indeed, as basement membrane is found in non-neural tissues, it is similarly lacking in the brain. However, because of the high expression of dystroglycans in the brain, additional ligands were sought. There is a strong association of both dystroglycan subunits with the neurexins (Sugita et al., 2001; Reissner et al., 2014). Neurexins are neuron-specific cell surface proteins, and although only three vertebrate neurexin genes have been identified, there are thought to be over a thousand alternative splice-generated forms (Ullrich et al., 1995). The neurexin structure can be complex in that it includes an extracellular domain consisting of a number of laminin-neurexin, sex steroid-binding globulin domains that are interspersed with EGF-like domains, although this region is variable depending upon the splice variant. Dystroglycans specifically bind to this region under the appropriate glycosylation and appears to compete with α-latrotoxin, as neurexin has been identified as an alternate viable receptor for α-latrotoxin (Sudhof, 2001; Sugita et al., 2001).

## Evidence for latrophilins as cognate receptors of teneurins and TCAPs

Several lines of evidence indicate that TCAP is an endogenous ligand of the LPHNs, and these arguments can be divided into evolution and phylogenetic, cellular expression, molecular and physiological aspects. At the evolutionary and phylogenetic level, TCAP appears to have evolved about the same time as the LPHNs at an early stage of metazoan evolution (Zhang et al., 2012; Chand et al., 2013; Lovejoy and de Lannoy, 2013). TCAP-like sequences are found in most metazoans and possess sequence similarity to a diverse range of hormones associated with the secretin GPCR family of ligands in vertebrates, and toxins found in both invertebrates and vertebrates. All TCAPs are associated, in part, with the expression of the teneurins. Our studies indicate that while mRNA expression of all TCAPs show a relationship with the intact teneurin, TCAP-1 is transcribed independently and TCAP-3 may also do so (Chand et al., 2013). The teneurin expression shows a strong overlap with the LPHNs, and TCAP and teneurins are found in all tissues known to express the LPHNs, including the amygdala, hippocampus, and prefrontal cortex (Arcos-Burgos et al., 2010; Silva et al., 2011). At the molecular level, teneurins have been established to bind to LPHNs at physiologically compatible affinity at the lectin-like domain (Silva et al., 2011; Boucard et al., 2012). This domain is the most highly conserved region of LPHNs, further indicating the ancestry and importance of teneurin-latrophilin binding.

## Neurological actions of the teneurin/TCAP-LPHN complex

There are a number of synaptic adhesion proteins, although only the teneurins, LPHNs and FLRTs have been conserved between invertebrates and vertebrates (Tucker et al., 2006; Boucard et al., 2014). Therefore, the teneurin-latrophilin pairing is a likely candidate to understand the phylogenetically earliest mechanism that lead to neuronal transmission. Interneuronal transmission is essential for neuron viabilities. Tan et al. (2011) showed that intracerebroventricular (ICV) administration of synthetic mTCAP-1 increased spine density in hippocampal neurons. Interestingly, LPHN-1 has been localized on nerve terminals near synaptic clefts but not on dendritic spines whereas teneurin-2 has been found on dendritic spines, suggesting that the two types of receptors may have different structure-function attributes (Silva et al., 2011). Also, as mentioned previously, post-synaptic FLRTs regulate glutamatergic synapse density in hippocampal cells; thus, providing support for their role in the trans-synaptic cell adhesion complex (O'Sullivan et al., 2014).

This interaction suggests a plausible mechanism for TCAP action: the LPHN-teneurin trans-synaptic complex stabilizes the synapse (along with the accessory proteins, dystroglycan, and neurexins) and the TCAP region of the C-terminal region of teneurin is required for high affinity binding to LPHN. Then, the presence of free TCAP, either in the synthetic form or in the form of a natural transcribed ligand, may bind to the hormone binding domain of LPHN and compete, in part, for the teneurin binding. Once bound, TCAP may regulate the actions/coupling of the receptor depending on the LPHN.

TCAP affects pathways related to anxiety and emotionality. Wang and associates screened rats by their response to the acoustic startle test, and then divided the rats into either low or high reactive groups based on their results (Wang et al., 2005). When treated with ICV TCAP-1, high reactive rats had a reduced acoustic startle response, whereas low reactive rats had an increased response, indicating that TCAP has a normalizing effect on behavior. It is proposed that TCAP has a general action across all synapses: synaptic circuits that promote a stress response would be disrupted leading to a decrease in stress behavior because of the inability to perceive and carry out the stress. In animals with a reduced sympathetic response and a higher parasympathetic response would be, likewise, inhibited and show a reduced inhibition of the stress response and so both would show a normalization of behavior. In the case of findings from Tan et al. (2011), the increase in spine density may reflect a normal homeostatic mechanism to increase the connections after synthetic TCAP-1 acted to break existing synapses. In the short term, the post-synaptic regions would be expected to increase dendrite spine number to maintain the signal. Then, in the long term and under repeated TCAP-1 administration (and assuming TCAP-1 reduces neurotransmitter release) the spine density would be reduced due to continued reduction of the neurotransmitter signal.

As part of the LPHN trans-synaptic complex, teneurins function to maintain neural networks by promoting synapse connectivity and increasing neurite outgrowth (Rubin et al., 1999; Young and Leamey, 2009). High levels of teneurins in the central nervous system are found on axon surfaces (Wang et al., 2005), and there is significant teneurin expression in the interconnected neurons found in chicken and mouse (Kenzelmann et al., 2008). Since the length of teneurin and LPHNs together can span the synaptic cleft (Silva et al., 2011), this allows teneurins to interact with adjacent neurons thus increasing efficiency of the synapse.

Additionally, teneurins are integral to the normal development of visual, auditory, and olfactory pathways (Young and Leamey, 2009; Hong et al., 2012). For example, teneurin-3 is required for binocular vision, as it regulates mapping of ipsilateral axons connecting the ventral retina to the dorsal lateral geniculate nucleus (Young and Leamey, 2009). Moreover, teneurin-1 is expressed prominently in the L5 and L6 regions of the neocortex (O'Sullivan et al., 2014) and in the piriform cortex, a region associated with processing of odor and pheromone signals (Wang et al., 2005). Ultimately, teneurins are important for basic brain maturation and function, as they regulate synaptic plasticity and modulate sensory processes.

A number of biological functions both *in vitro* and *in vivo* have been attributed to the TCAP family of peptides. The first studies found that in immortalized mouse neurons, TCAP-1 treatment had a dose-dependent effect on cAMP levels, teneurin-1 gene expression and cell proliferation (Qian et al., 2004; Wang et al., 2005; Al Chawaf et al., 2007b). Further, TCAP-1 is neuroprotective in hypothalamic neurons by increasing superoxide dismutase-1, superoxide dismutase-1 copper chaperone and catalase enzyme levels (Trubiani et al., 2007). In unstressed hypothalamic cells and primary hippocampal neurons, TCAP-1 treatment increases expression of α-actinin-4, β-actin and β-tubulin; and also induced neurite outgrowth, dendritic arborization, and axon fasciculation (Al Chawaf et al., 2007a). TCAP-1 also inhibits brain-derived neurotrophic factor expression and translation in hypothalamic neurons (Anantharaman and Aravind, 2003).

The receptor complex and signal transduction system for TCAP-1 has been recently established. Gene array studies initially established that the β-dystroglycan gene was significantly upregulated in TCAP-1 treated immortalized hypothalamic neurons. Immunohistochemical studies established that FITC-tagged synthetic TCAP-1 strongly co-localized with β-dystroglycan at the plasma membrane. In addition, the MEK-ERK1/2 signal transduction system associated with β-dystroglycan was, likewise, stimulated by TCAP-1 and could be blocked by a MEK inhibitor (Chand et al., 2012b) Subsequently, TCAP-1 stimulated ERK1/2-dependent phosphorylation of the cytoskeleton regulatory proteins, filamin, and stathmin. These studies indicate that TCAP-1 signals through a novel pathway associated with the dystroglycan complex to regulate cytoskeletal dynamics. Moreover, it provides an understanding of how TCAP and the teneurins can regulate neurite and neuronal process formation. Recently, studies on the carboxy-terminal region of teneurins on cytoskeletal elements in *Drosophila* showed a similar mechanism (Hong et al., 2012; Mosca et al., 2012). The two *Drosophila* teneurin proteins, ten-m and ten-a, have been implicated in neuromuscular synapse organization. Following teneurin perturbation, proper microtubule organization was impaired at presynaptic terminals, and post-synaptic spectrin cytoskeleton, α- and β-spectrin, an essential component of the membraneous subsynaptic reticulum, was significantly reduced. The most severe effects seen were cytoskeletal, thus, teneurins and TCAP play a vital role in pre-synaptic and post-synaptic cytoskeleton organization in *Drosophila*. (Hong et al., 2012; Mosca et al., 2012).

## Cognitive and behavioral effects of the teneurin/TCAP-LPHN system

Studies of the LPHNs have indicated a number of neurological effects specifically in the limbic region, and recent work has aimed at elucidating the neuronal expression of LPHNs and their role in psychiatric disorders. For instance, high *Lphn1* expression was found in the rat hippocampus and high *LPHN3* mRNA expression was found in human mesolimbic brain regions such as the amygdala and prefrontal cortex (Arcos-Burgos et al., 2010; Silva et al., 2011). *Lphn1* knockout studies have revealed some of its neurological actions. Female mice who were homozygous null mutants for *Lphn1* were not able to attend to their litters compared to wild-type mice, resulting in the death of their pups regardless of the genotype of the pups themselves (Martinez et al., 2010). As well, Silva and Ushkaryov (2010) found that *Lphn1* knockout mice showed increased aggression and schizophrenia-like behaviors compared to wild-type mice.

High *lphn3* expression was also examined in zebrafish dopamine neurons in the posterior tuberculum, a brain area with similar functions as the mammalian ventral tegmental area (Lange et al., 2012). Furthermore, loss of the *Lphn3* gene has been implicated in impaired dopamine system development and the pathogenesis of cocaine addiction and attention-deficient hyperactive disorder (ADHD) (Lange et al., 2012; Wallis et al., 2012). A hyperactive motor phenotype was seen in *lphn3*-mutant zebrafish, as indicated by their fast swimming behavior (Lange et al., 2012). Moreover, mutated *LPHN3* gene may be implicated in developmental synaptic abnormalities underlying ADHD (O'Sullivan et al., 2014). Similar to FLRTs, LPHN-3 controls glutamatergic synapse development and abundance of synaptic inputs in mouse pyramidal neurons. It is suggested that a mutation in the lectin domain of LPHN3 impairs binding of teneurin-1 to LPHN in the mouse neocortex (O'Sullivan et al., 2014). Interestingly, rats that repeatedly self-administered cocaine show upregulated *Lphn1* levels in the striatum (Lynch et al., 2008). Thus, LPHNs may be important components in dopamine-mediated mechanisms and emotional regulation. Interestingly, GPCRs have been of major pharmaceutical interest, with about one-third of research being directed at them (Martinez et al., 2010). This further supports the importance of this research of the Teneurin/TCAP-LPHN ligand-receptor interaction and its medicinal applications in respect to neurological conditions. Further studies indicate that the teneurin/TCAP-latrophilin system may have a number of effects on cell and organismal metabolism.

TCAP-1 has emerged as a novel candidate in the integration and modulation of a number of psychiatric disorders including stress, anxiety, and addiction (Tan et al., 2008). Acute administration of TCAP-1 into the basolateral nucleus of the amygdala in rats modulated acoustic startle behavior by increasing the startle response in the low-startle group and decreasing startle response in the high-startle group (Wang et al., 2005). Other anxiety studies, using an elevated plus maze and open field tests, showed that TCAP-1 modulates CRF-regulated behaviors. Repeated intravenous (IV) administration of TCAP-1 had an anxiolytic effect on CRF-induced responses in both the elevated plus maze and open field tests (Al Chawaf et al., 2007b). However, ICV administration of TCAP-1 yielded differential behavioral responses based on the presence or absence of a stress challenge (Tan et al., 2008). In the absence of a CRF-mediated stressor, TCAP-1 had mild anxiolytic effects in the elevated plus maze and open field tests of behavior however, in the presence of a stressor TCAP-1 had anxiogenic effects in the elevated plus maze and open field tests (Tan et al., 2008). Interestingly, the TCAP-1 induced effects on behavior are long lasting, with effects persisting 21 days after TCAP-1 injection (Wang et al., 2005), suggesting that TCAP-1 may regulate neuronal plasticity in the brain. Further *in vivo* studies strengthened the notion of a functional link between the TCAP-1 and CRF-mediated systems. TCAP-1 blocked CRF-mediated c-fos synthesis in the hippocampus and amygdala of the adult rat (Tan et al., 2009) and increased spine density in the CA1 and CA3 neurons of the rodent hippocampus but not in the amygdala (Tan et al., 2011). Moreover, repeated ICV and IV TCAP- 1 administration ablates CRF-induced cocaine-seeking reinstatement in rats (Kupferschmidt et al., 2011; Erb et al., 2014).

## Summary

As indicated by evolutionary and phylogenetic, molecular and physiological aspects, we posit that TCAP is the endogenous ligand for LPHN. This cognate ligand-receptor coupling has been retained since their ancient interaction approximately one billion years ago. Endogenous TCAP-teneurin is bound to a trans-synaptic LPHN cell adhesion complex and is associated with FLRTs, neurexins, and dystroglycans. Ultimately, this complex is integral to maintenance of synaptic plasticity and regulating pathways associated with behavior. While little is known about the mechanisms behind the expression of TCAP-1, evidence strongly suggests that it can be transcribed independently of teneurin-1 and is translated and processed outside of the classical ER/Golgi pathway. This, as well as its distinct functions and cellular expression compared to teneurin-1, indicate that the two have independent roles within the cell.

### Conflict of interest statement

Dr. D. A. Lovejoy is the Chief Scientific Officer of Protagenic Therapeutics, Inc (PTI). The authors declare that the research was conducted in the absence of any commercial or financial relationships that could be construed as a potential conflict of interest.

## References

[B1] Al Chawaf A. St. Amant K. Belsham D. D. Lovejoy D. A. (2007a). Regulation of neurite outgrowth in immortalized hypothalamic cells and hippocampal primary cultures by teneurin C-terminal associate peptide-1 (TCAP-1). Neuroscience 144, 1241–1254. 10.1016/j.neuroscience.2006.09.062 17174479

[B2] Al Chawaf A. Xu K. Tan L. Vaccarino F. Lovejoy D. A. Rotzinger S. (2007b). Corticotropin-releasing factor behaviours are modulated by intravenous administration of teneurin C-terminal associated peptides. Peptides 28, 1406–1415. 10.1016/j.peptides.2007.05.014 17644218

[B3] Anantharaman V. Aravind L. (2003). New connections in the prokaryote toxin-antitoxin network: relationship with the eukaryote nonsense-mediated RNA decay system. Genome Biol. 4:R81. 10.1186/gb-2003-4-12-r81 14659018 PMC329420

[B4] Arcos-Burgos M. Jain M. Acosta M. T. Shively S. Stanescu H. Wallis D.. (2010). A common variant of the latrophilin 3 gene, *Lphn3*, confers susceptibility to ADHD and predicts effectiveness of stimulant medication. Mol. Psychiatr. 15, 1053–1066. 10.1038/mp.2010.6 20157310

[B5] Banks G. Fuhrer C. Adams M. Froehner S. (2003). The postsynaptic submembrane machinery at the neuromuscular junction: requirement for rapsyn and the utrophin/dystrophin-associated complex. J. Neurocytol. 32, 709–726. 10.1023/B:NEUR.0000020619.24681.2b 15034263

[B6] Baumgartner S. Chiquet-Ehrismann R. (1993). Ten(a), a Drosophila gene related to tenascin, shows selective transcript localization. Mech. Dev. 40, 165–176. 10.1016/0925-4773(93)90074-8 7684246

[B7] Baumgartner S. Martin D. Hagios C. Chiquet-Ehrismann R. (1994). Tenm, a Drosophila gene related to tenascin, is a new pair-rule gene. EMBO J. 13, 3728–3740. 8070401 10.1002/j.1460-2075.1994.tb06682.x PMC395283

[B8] Boucard A. Ko J. Sudhof T. (2012). High affinity neurexin binding to cell adhesion G-protein-coupled receptor CIRL1/latrophilin-1 produces an intracellular adhesion complex. J. Biol. Chem. 287, 9399–9413. 10.1074/jbc.M111.318659 22262843 PMC3308797

[B9] Boucard A. A. Maxeiner S. Südhof T. C. (2014). Latrophilins function as heterophilic cell-adhesion molecules by binding to teneurins: regulation by alternative splicing. J. Biol. Chem. 289, 389–402. 10.1074/jbc.M113.504779 24273166 PMC3879561

[B10] Boudier J. A. Martin-Moutot N. Boudier J. L. Iborra C. Takahashi M. Seagar M. J. (1999). Redistribution of presynaptic proteins during α-latrotoxin-induced release of neurotransmitter and membrane retrieval at the frog neuromuscular junction. Eur. J. Neurosci. 11, 3449–3456. 10.1046/j.1460-9568.1999.00778.x 10564353

[B11] Chand D. Casatti C. A. de Lannoy L. Song L. Kollara A. Barsyte-Lovejoy D.. (2012a). C-terminal processing of the teneurin proteins: independent actions of a teneurin C-terminal associated peptide in hippocampal cells. Mol. Cell. Neurosci. 52, 38–50. 10.1016/j.mcn.2012.09.006 23026563

[B12] Chand D. Colacci M. Dixon K. Kollara A. Brown T. J. Lovejoy D. A. (2014). C-terminal region of teneurin-1 co-localizes with the dystroglycan complex in adult mouse testes and regulates testicular size and testosterone production. Histochem. Cell Biol. 141, 191–211. 10.1007/s00418-013-1154-1 24154551

[B13] Chand D. de Lannoy L. Tucker R. Lovejoy D. A. (2013). Origin of chordate peptides by horizontal protozoan gene transfer in early metazoans and protists: evolution of the teneurin C-terminal associated peptides. Gen. Comp. Endo. 188, 144–150. 10.1016/j.ygcen.2013.02.006 23453965

[B14] Chand D. Song L. de Lannoy L. Barsyte-Lovejoy D. Ackloo S. Boutros P. C.. (2012b). C-terminal region of teneurin-1 co-localizes with dystroglycan and modulates cytoskeletal organization through an ERK-dependent stathmin- and filamin A-mediated mechanism in hippocampal cells. Neuroscience 219, 255–270. 10.1016/j.neuroscience.2012.05.069 22698694

[B15] Davydov I. I. Fidalgo S. Khaustova S. A. Lelyanova V. G. Grebenyuk E. S. Ushkaryov Y. A.. (2009). Prediction of epitopes in closely related protein using a new algorithm. Bull. Exp. Biol. Med. 148, 869–873. 10.1007/s10517-010-0838-y 21116493

[B16] Drabikowski K. Trzebiatowska A. Chiquet-Ehrismann R. (2005). ten-1, an essential gene for germ cell development, epidermal morphogenesis, gonad migration, and neuronal pathfinding in *Caenorhabditis elegans*. Dev. Biol. 282, 27–38. 10.1016/j.ydbio.2005.02.017 15936327

[B17] Erb S. McPhee M. Brown Z. J. Kupferschmidt D. A. Song L. Lovejoy D. A. (2014). Repeated intravenous administrations of teneurin C-terminal associated peptide (TCAP-1) attenuates reinstatement of cocaine seeking by corticotropin-releasing factor (CRF) in rats. Behav. Brain Res. 269, 1–5. 10.1016/j.bbr.2014.04.013 24768621

[B18] Fredricksson R. Lagerstrom M. C. Lundin L. G. Schioth H. B. (2003). The G-protein-coupled receptors in the human genome form five main families. Phylogenetic analysis, paralogon groups, and fingerprints. Mol. Pharmacol. 63, 1256–1272. 10.1124/mol.63.6.1256 12761335

[B19] Hemler M. E. (1999). Dystroglycan versatility. Cell 97, 543–546. 10.1016/S0092-8674(00)80764-3 10367882

[B20] Henry M. Campbell K. (1996). Dystroglycan: an extracellular matrix receptor linked to the cytoskeleton. Curr. Opin. Cell Biol. 8, 625–631. 10.1016/S0955-0674(96)80103-7 8939660

[B21] Henry M. Campbell K. (1998). A role for dystroglycan in basement membrane assembly. Cell 95, 859–870. 10.1016/S0092-8674(00)81708-0 9865703

[B22] Henry M. Campbell K. (1999). Dystroglycan inside and out. Curr. Opin. Cell Biol. 11, 602–607. 10.1016/S0955-0674(99)00024-1 10508656

[B23] Henry M. D. Satz J. S. Brakebusch C. Costell M. Gustafsson E. Fassler R.. (2001). Distinct roles for dystroglycan, β1 integrin and perlecan in cell surface laminin organization. J. Cell Sci. 114, 1137–1144. 11228157 10.1242/jcs.114.6.1137

[B24] Hong W. Mosca T. J. Luo L. (2012). Teneurins instruct synaptic partner matching in an olfactory map. Nature 484, 201–207. 10.1038/nature10926 22425994 PMC3345284

[B25] Kenzelmann D. Chiquet-Ehrismann R. Leachman N. T. Tucker R. P. (2008). Teneurin-1 is expressed in interconnected regions of the developing brain and is processed *in vivo*. BMC Dev. Biol. 8:30. 10.1186/1471-213X-8-30 18366734 PMC2289808

[B26] Kupferschmidt D. A. Lovejoy D. A. Rotzinger S. Erb S. (2011). Teneurin C-terminal associated peptide-1 blocks the effects of corticotropin-releasing factor on reinstatement. Brit. J. Pharmacol. 162, 574–583 10.1111/j.1476-5381.2010.01055.x 20883474 PMC3041248

[B27] Lange M. Norton W. Coolen M. Chaminade M. Merker S. Proft F.. (2012). The ADHD-susceptibility gene *lphn3.1* modulates dopaminergic neuron formation and locomotor activity during zebrafish development. Mol. Psychiatr. 17, 946–954. 10.1038/mp.2012.29 22508465

[B28] Lelianova V. Davletov B. Sterling A. Rahman M. A. Grishin E. Totty N.. (1997). α-latrotoxin receptor, latrophilin, is a novel member of the secretin family of G protein-coupled receptors. J. Biol. Chem. 272, 21504–21508. 10.1074/jbc.272.34.21504 9261169

[B29] Lelyanova V. Thomson D. Ribchester R. R. Tonevitsky E. A. Ushkaryov Y. A. (2009). Activation of α-latrotoxin receptors in neuromuscular synapses leads to a prolonged splash of acetylcholine release. Exp. Biol. Med. 147, 701–703. 10.1007/s10517-009-0600-5 19902061

[B30] Levine A. Bashan-Ahrend A. Budai-Hadrian O. Gartenberg D. Menasherow S. Wides R. (1994). Odd Oz: a novel Drosophila pair rule gene. Cell 77, 587–598. 10.1016/0092-8674(94)90220-8 7514504

[B31] Lossie A. C. Nakamura H. Thomas S. E. Justice M. J. (2005). Mutation of l7Rn3 shows that Odz4 is required for mouse gastrulation. Genetics 169, 285–299. 10.1534/genetics.104.034967 15489520 PMC1448887

[B32] Lovejoy D. A. Al Chawaf A. Cadinouche A. (2006). Teneurin C-terminal associated peptides: an enigmatic family of neuropeptides with structural similarity to the corticotrophin releasing factor and calcitonin family of peptides. Gen. Comp. Endocrinol. 148, 299–305. 10.1016/j.ygcen.2006.01.012 16524574

[B33] Lovejoy D. A. de Lannoy L. (2013). Evolution and phylogeny of the corticotropin releasing factor (CRF) family of peptides: expansion and specialization in the vertebrates. J. Chem. Neuroanat. 54, 50–56. 10.1016/j.jchemneu.2013.09.006 24076419

[B34] Lynch W. J. Girgenti J. Breslin F. J. Newton S. S. Taylor J. R. (2008). Gene profiling the response to repeated cocaine self-administration in dorsal striatum: a focus on circadian genes. Brain Res. 1213, 166–177. 10.1016/j.brainres.2008.02.106 18452895 PMC2494701

[B35] Martinez A. F. Muenke M. Arcos-Burgos M. (2010). From the Black Widow spider to human behaviour: latrophilins, a relatively unknown class of G protein-coupled receptors, are implicated in psychiatric disorders. Am. J. Med. Genet. B 156, 1–10 10.1002/ajmg.b.31137 PMC4101183 21184579

[B36] Masaki T. Matsumura K. (2010). Biological role of dystroglycan in Schwann cell function and its implications in peripheral nervous system diseases. J. Biomed. Biothechnol. 2010:740403. 10.1155/2010/740403 20625412 PMC2896880

[B37] Matsushita H. Lelianova V. G. Ushkaryov Y. A. (1999). The latrophilin family: multiply spliced G protein-coupled receptors with differential tissue distribution. FEBS Letters. 443, 348–352. 10.1016/S0014-5793(99)00005-8 10025961

[B38] Mieda M. Kikuchi Y. Hirate Y. Aoki M. Okamoto H. (1999). Compartmentalized expression of zebrafish ten-m3 and ten-m4, homologues of the Drosophila ten(m)/odd Oz gene, in the central nervous system. Mech. Dev. 87, 223–227. 10.1016/S0925-4773(99)00155-0 10495292

[B39] Minet A. D. Chiquet-Ehrismann R. (2000). Phylogenetic analysis of teneurin genes and comparison to the rearrangement hot spot elements of *E. coli*. Gene 257, 87–97. 10.1016/S0378-1119(00)00388-7 11054571

[B40] Minet A. D. Rubin B. P. Tucker R. P. Baumgartner S. Chiquet-Ehrismann R. (1999). Teneurin-1, a vertebrate homologue of the Drosophila pair-rule gene ten-m, is a neuronal protein with a novel type of heparin-binding domain. J. Cell. Sci. 112, 2019–2032. 10341219 10.1242/jcs.112.12.2019

[B41] Mosca T. J. Hong W. Dani V. S. Favaloro V. Luo L. (2012). Trans-synaptic Teneurin signalling in neuromuscular synapse organization and target choice. Nature 484, 237–241. 10.1038/nature10923 22426000 PMC3326183

[B42] Ni T. Yue J. Sun G. Zou Y. Wen J. Huang J. (2012). Ancient gene transfer from algae to animals: mechanisms and evolutionary significance. BMC Mol. Biol. 12:83. 10.1186/1471-2148-12-83 22690978 PMC3494510

[B43] Nordstrom K. Lagerstrom M. Waller L. Fredriksson R. Schioth H. (2009). The secretin GPCRs descended from the family of adhesion GPCRs. Mol. Biol. Evol. 26, 71–84. 10.1093/molbev/msn228 18845549

[B44] Nunes S. M. Ferralli J. Choi K. Brown-Luedi M. Minet A. D. Chiquet-Ehrismann R. (2005). The intracellular domain of teneurin-1 interacts with MBD1 and CAP/ponsin resulting in subcellular codistribution and translocation to the nuclear matrix. Exp. Cell Res. 305, 122–132. 10.1016/j.yexcr.2004.12.020 15777793

[B45] O'Sullivan M. L. De Wit J. Savas J. N. Comoletti D. Otto-Hitt S. Yates J. R. III. (2012). FLRT proteins are endogenous latrophilin ligands and regulate excitatory synapse development. Neuron 73, 903–910. 10.1016/j.neuron.2012.01.018 22405201 PMC3326387

[B46] O'Sullivan M. L. Martini F. von Daake S. Comoletti D. Ghosh A. (2014). LPHN3, a pre-synaptic adhesion-GPCR implicated in ADHD regulates the strength of neocortical layer 2/3 synaptic input to layer 5. Neural Dev. 9:7. 10.1186/1749-8104-9-7 24739570 PMC3996519

[B47] Oohashi T. Zhou X. H. Feng K. Richter B. Morgelin M. Perez M. T.. (1999). Mouse ten-m/Odz is a new family of dimeric type II transmembrane proteins expressed in many tissues. J. Cell Biol. 145, 563–577. 10.1083/jcb.145.3.563 10225957 PMC2185078

[B48] Otaki J. M. Firestein S. (1999). Neurestin: putative transmembrane molecule implicated in neuronal development. Dev. Biol. 212, 165–181. 10.1006/dbio.1999.9310 10419693

[B49] Perrin M. H. Sutton S. Bain D. L. Berggren W. T. Vale W. W. (1998). The first extracellular domain of corticotropin releasing factor-R1 contains major binding determinants for urocortin and adstressin. Endocrinology 139, 566–570. 10.1210/en.139.2.566 9449626

[B50] Qian X. Barsyte-Lovejoy D. Chewpoy R. B. Wang L. Gautam N. Wang N.. (2004). Characterization of teneurin C-terminal associated peptide (TCAP)-3 from rainbow trout hypothalamus. Gen. Comp. Endocrinol. 137, 205–216. 10.1016/j.ygcen.2004.02.007 15158132

[B51] Ramulu H. G. Raoult D. Pontarotti P. (2012). The rhizome of life: what about metazoan? Cell. Infect. Microbiol. 2, 1–11 10.3389/fcimb.2012.00050 PMC3417402 22919641

[B52] Reissner C. Stahn J. Breuer D. Klose M. Pohlentz G. Mormann M.. (2014). Dystroglycan binding to α-neurexin competes with neurexophilin-1 and neuroligin in the brain. J. Biol. Chem. 289, 27585–27603. 10.1074/jbc.M114.595413 25157101 PMC4183798

[B53] Rubin B. P. Tucker R. P. Brown-Luedi M. Martin D. Chiquet-Ehrismann R. (2002). Teneurin 2 is expressed by the neurons of the thalamofugal visual system *in situ* and promotes homophilic cell-cell adhesion *in vitro*. Development 129, 4697–4705. 12361962 10.1242/dev.129.20.4697

[B54] Rubin B. P. Tucker R. P. Martin D. Chiquet-Ehrismann R. (1999). Teneurins: a novel family of neuronal cell surface proteins in vertebrates, homologous to the *Drosophila* pair-rule gene product Ten-m. Dev. Biol. 216, 195–209. 10.1006/dbio.1999.9503 10588872

[B55] Schiöth H. B. Nordström K. J. Fredriksson R. (2010). The adhesion GPCRs: gene repertoire, phylogeny and evolution. Adv. Exp. Med. Biol. 706, 1–13. 10.1007/978-1-4419-7913-1_1 21618822

[B56] Silva J. P. Leilanova V. G. Ermolyuk Y. S. Vysokov N. Hitchen P. G. Berninghausen O.. (2011). Latrophilin 1 and its endogenous ligand Lasso/teneurin-2 form a high-affinity transsynaptic receptor pair with signaling capabilities. Proc. Natl. Acad. Sci. U.S.A. 108, 12113–12118. 10.1073/pnas.1019434108 21724987 PMC3141932

[B57] Silva J. P. Ushkaryov Y. A. (2010). The latrophilins, “split personality” receptors. Adv. Exp. Med. Biol. 706, 59–75. 10.1007/978-1-4419-7913-1_5 21618826 PMC3145135

[B58] Storchak L. G. Linetska M. V. Himmelreich N. H. (2002). Does extracellular calcium determine what pool of GABA is the target for α-latrotoxin? Neurochem. Int. 40, 387–395. 10.1016/S0197-0186(01)00107-3 11821145

[B79] Sudhof T. C. (2001). α-latrotoxin and its receptors: neurexins and Cirl/latrophilins. Annu. Rev. Neurosci. 24, 933–962. 10.1146/annurev.neuro.24.1.933 11520923

[B59] Sugita S. Saito F. Tang J. Satz J. Campbell K. Sudhof T. (2001). A stoichiometric complex of neurexins and dystroglycan in brain. J. Cell Biol. 154, 435–445. 10.1083/jcb.200105003 11470830 PMC2150755

[B60] Tan L. A. Al Chawaf A. Vaccarino F. J. Boutros J. C. Lovejoy D. A. (2011). Teneurin C-terminal associated peptide (TCAP)-1 increases dendritic spine density in hippocampal neurons and decreases anxiety-like behaviors in rats. Physiol. Behav. 104, 199–204. 10.1016/j.physbeh.2011.03.015 21411044

[B61] Tan L. Xu K. Vaccarino F. Lovejoy D. A. Rotzinger S. (2008). Repeated intracerebral teneurin C-terminal associated peptide (TCAP)-1 injections produce enduring changes in behavioral responses to corticotropin-releasing factor (CRF) in rat models of anxiety. Behav. Brain Res. 188, 195–200. 10.1016/j.bbr.2007.10.032 18082275

[B62] Tan L. Xu K. Vaccarino F. J. Lovejoy D. A. Rotzinger S. (2009). Teneurin C-terminal associated peptide (TCAP)-1 attenuates corticotropin-releasing factor (CRF)-induced c-Fos expression in the limbic system and modulates anxiety behaviour in male Wistar rats. Behav. Brain Res. 201, 198–206. 10.1016/j.bbr.2009.02.013 19428634

[B63] Tan L. A. Chand D. De Almeida R. Xu M. Colacci M. de Lannoy L.. (2012). Modulation of neuroplastic changes and corticotropin-releasing factor associated behaviour by a phylogenetically ancient and conserved peptide family. Gen. Comp. Endocrinol. 176, 309–313. 10.1016/j.ygcen.2011.11.011 22138219

[B64] Trubiani G. Al Chawaf A. Belsham D. D. Barsyte-Lovejoy D. Lovejoy D. A. (2007). Teneurin carboxy (C)-terminal associated peptide-1 inhibits alkalosis-associated necrotic cell death by stimulating superoxide dismutase and catalase activity in immortalized mouse hypothalamic cells. Brain Res. 1176, 27–36. 10.1016/j.brainres.2007.07.087 17900539

[B65] Trzebiatowska A. Topf U. Sauder U. Drabikowski K. Chiquet-Ehrismann R. (2008). *Caenorhabditis elegans* teneurin, *ten-1*, is required for gonadal and pharyngeal basement membrane integrity and acts redundantly with integrin *ina-1* and dystroglycan *dgn-1*. Mol. Biol. Cell 19, 3898–3908. 10.1091/mbc.E08-01-0028 18632986 PMC2526705

[B66] Tucker R. P. (2013). Horizontal gene transfer in choanoflagellates. J. Exp. Zool. B Mol. Dev. Evol. 320, 1–9. 10.1002/jez.b.22480 22997182

[B67] Tucker R. P. Beckmann J. Leachman N. T. Scholer J. Chiquet-Ehrismann R. (2012). Phylogenetic analysis of the teneurins: conserved features and premetazoan ancestry. Mol. Biol. Evol. 29, 1019–1029. 10.1093/molbev/msr271 22045996 PMC3278476

[B68] Tucker R. P. Chiquet-Ehrismann R. (2006). Teneurins: a conserved family of transmembrane proteins involved in intercellular signaling during development. Dev. Biol. 290, 237–245. 10.1016/j.ydbio.2005.11.038 16406038

[B69] Tucker R. P. Drabikowski K. Hess J. F. Ferralli J. Chiquet-Ehrismann R. Adams J. C. (2006). Phylogenetic analysis of the tenascin gene family: evidence of origin early in the chordate lineage. BMC Evol. Biol. 6:60. 10.1186/1471-2148-6-60 16893461 PMC1578592

[B70] Ullrich B. Ushkaryov Y. A. Südhof T. C. (1995). Cartography of neurexins: more than 1000 isoforms generated by alternative splicing and expressed in distinct subsets of neurons. Neuron 14, 497–507. 10.1016/0896-6273(95)90306-2 7695896

[B71] Vakonakis I. Langenhan T. Prömel S. Russ A. Campbell I. D. (2008). Solution structure and sugar-binding mechanism of mouse latrophilin-1 RBL: a 7TM receptor-attached lectin-like domain. Structure 16, 944–953. 10.1016/j.str.2008.02.020 18547526 PMC2430599

[B72] Wallis D. Hill D. S. Mendez I. A. Abbott L. C. Finnell R. H. Wellman P. L.. (2012). Initial characterization of mice null for *Lphn3*, a gene implicated in ADHD and addiction. Brain Res. 1463, 85–92. 10.1016/j.brainres.2012.04.053 22575564

[B73] Wang L. Rotzinger S. Al Chawaf A. Elias C. F. Barsyte-Lovejoy D. Qian X. (2005). Teneurin proteins possess a carboxy terminal sequence with neuromodulatory activity. Mol. Brain Res. 133, 253–265 10.1016/j.molbrainres.2004.10.019 15710242

[B74] Wang X. Z. Kuroda M. Sok I. Batchvarova N. Kimmel R. Chung P.. (1998). Identification of novel stress-induced genes downstream of chop. EMBO J. 17, 3619–3630. 10.1093/emboj/17.13.3619 9649432 PMC1170698

[B75] Williamson R. Henry M. Daniels K. Hrstka R. Lee J. Sunada Y.. (1997). Dystroglycan is essential for early embryonic development: disruption of Reichert's membrane in Dag1-null mice. Hum. Molec. Genet. 6, 831–841. 10.1093/hmg/6.6.831 9175728

[B76] Young T. R. Leamey C. A. (2009). Teneurins: important regulators of neural circuitry. Int. J. Biochem. Cell. Biol. 41, 990–993. 10.1016/j.biocel.2008.06.014 18723111

[B77] Zhang D. deSouza R. F. Anantharaman V. Iyer L. M. Aravind L. (2012). Polymorphic toxin systems: comprehensive characterization of trafficking modes, mechanism of action, immunity and ecology using comparative genomics. Biol. Direct. 7:18. 10.1186/1745-6150-7-18 22731697 PMC3482391

[B78] Zhou X. H. Brandau O. Feng K. Oohashi T. Ninomiya Y. Rauch U.. (2003). The murine Ten-m/Odz genes show distinct but overlapping expression patterns during development and in adult brain. Gene Expr. Patterns 3, 397–405. 10.1016/S1567-133X(03)00087-5 12915301

